# Three Cases of West Nile Encephalitis over an Eight-Day Period at a Downtown Los Angeles Community Hospital

**DOI:** 10.1155/2015/262698

**Published:** 2015-05-28

**Authors:** Adam Puchalski, Antonio K. Liu, Byron Williams

**Affiliations:** White Memorial Medical Center, 1720 E. Cesar Chavez Avenue, Los Angeles, CA 90033, USA

## Abstract

Since its introduction in New York City in 1999, the virus has spread throughout the entire North American continent and continues to spread into Central and Latin America. Our report discusses the signs and symptoms, diagnostics, and treatment of West Nile disease. It is important to recognize the disease quickly and initiate appropriate treatment. We present three cases of West Nile encephalitis at White Memorial Medical Center in East Los Angeles that occurred over the span of eight days. All three patients live within four to six miles from the hospital and do not live or work in an environment favorable to mosquitoes including shallow bodies of standing water, abandoned tires, or mud ruts. All the patients were Hispanic. Physicians and other health care providers should consider West Nile infection in the differential diagnosis of causes of aseptic meningitis and encephalitis, obtain appropriate laboratory studies, and promptly report cases to public health authorities. State governments should establish abatement programs that will eliminate sources that allow for mosquito reproduction and harboring. The public needs to be given resources that educate them on what entails the disease caused by the West Nile virus, what the symptoms are, and, most importantly, what they can do to prevent themselves from becoming infected.

## 1. Introduction

West Nile is an arbovirus that belongs to the Flaviviridae family. It is spread by the* Culex *mosquitoes that have bitten infected animals. It has usually been found in Africa, Europe, the Middle East, and Asia. West Nile was first introduced in the United States in 1999. There was an outbreak of meningitis and encephalitis in the New York City borough of Queens [1–3]. The virus has spread and is found across the United States as well as Canada, Mexico, the Caribbean, and Latin America. There are between 1000 and 3000 cases per year in the United States, resulting in approximately 100–300 deaths [1].

We present three cases of West Nile encephalitis at White Memorial Medical Center in East Los Angeles that occurred over the span of eight days. All three cases live within four to six miles from the hospital and do not live in an environment favorable to mosquitoes.

## 2. Cases

The first case was a 62-year-old male with a past medical history of diabetes mellitus, hypertension, and obesity who presented with altered mental status, lower extremity weakness, and falling. On physical examination, patient was confused, had a stiff neck, and had 0/5 strength in the left lower extremity. MRIs of the brain and spine were done, which were both negative for any spinal and intracranial pathology such as hyperdense lesions or tumors. CSF studies showed a white blood cell count of 228 with 79% polymorphonuclear cells and 17% lymphocytes, a protein count of 57, and a glucose count of 87. West Nile antibodies in the CSF were detected via ELISA and the results were as follows: IgM 4.83 (normal range: <0.89) and IgG 0.07 (normal range: <1.29). The previously mentioned studies were performed on the day of admission. Serum West Nile antibodies were performed via the ELISA two weeks later and the results were as follows: IgG 2.83 (normal range: <1.29) and IgM 7.37 (normal range: <0.89). His flaccid paralysis did not improve after 30 days.

The next case was a 62-year-old female with a past medical history of diabetes mellitus and hypertension who presented with headache, nausea, vomiting, and altered mental status of one-week duration. Per family, the patient was also incoherent. Her motor examination was within normal limits. Both MRI and EEG were negative for any pathology. CSF studies showed a white blood cell count of 105 with 1% polymorphonuclear cells and 93% lymphocytes, a protein count of 201, and glucose count of 67. West Nile antibodies in the CSF were detected via ELISA and the results were as follows: IgM 5.43 (normal range: <0.89) and IgG 2.81 (normal range: <1.29). The previously mentioned studies were performed on the day of admission. Serum West Nile antibodies were performed via the ELISA two weeks later and the results were as follows: IgG 5.26 (normal range: <1.29) and IgM 3.57 (normal range: <0.89).

The third case was a 59-year-old healthy female who presented with headache of one-week duration. She denied any change in mental status or weakness. Her neurological exam was nonfocal and only demonstrated headache. CT scan of the brain was negative for bleed or tumors. CSF studies showed a white blood cell count of 375 with 53% polymorphonuclear cells and 43% lymphocytes, a protein count of 101, and glucose count of 84. West Nile antibodies in the CSF were detected via ELISA and the results were as follows: IgG 1.26 (normal range: <1.29) and IgM 5.87 (normal range: <0.89). Patient recovered well and was discharged from the hospital after five days of supportive care.

## 3. Clinical Manifestations

The majority of patients infected with West Nile are completely asymptomatic. Symptomatic patients present with flu-like symptoms such as fatigue, maculopapular rash, headache, and gastrointestinal symptoms, which last for approximately one week [1, 3, 4].

Others can have more severe symptoms, such as neuroinvasive disease. These include encephalitis or meningitis with accompanying dramatic motor paresis or paralysis. Currently, it is believed that 1 : 150 patients infected with West Nile will have such symptoms [1, 3]. These symptoms are more common among elderly, diabetic, and hypertensive patients as well as among patients with previous CNS insults [1].

Approximately 15% of patients who have encephalitis progress to coma. Other abnormalities that may occur concomitantly include depressed DTRs, profound proximal muscle weakness, flaccid paralysis, and respiratory failure [2, 5]. EMG results in patients who had muscle weakness resembled a motor axonal polyneuropathy with sparing of the sensory fibers. However, one case did demonstrate demyelination polyneuritis, which exhibited a Guillain-Barre-like picture [5]. CSF findings include pleocytosis with a predominance of lymphocytes, elevated protein, and normal glucose levels.

Other neurological manifestations of West Nile, which are quite rare, include myelitis, optic neuritis, rhombencephalitis, and polyradiculitis.

## 4. Diagnosis

The most sensitive test used for diagnosis of West Nile is the serum or CSF IgM. There is some cross-reactivity with other IgM assays including St. Louis encephalitis, Dengue, and Yellow Fever, resulting in a low specificity of this test. As a result, IgG must be present in the same sample to confirm the diagnosis [2].

## 5. Treatment and Prevention

All patients with suspected West Nile meningitis or encephalitis should be brought to the hospital for supportive treatment and observation and to rule out treatable CNS illnesses such as herpesvirus, bacterial meningitis, or Guillain-Barre syndrome [5]. The most common cause of death is neuronal dysfunction, respiratory failure, and cerebral edema. The edema is a result of neuronal injury and death [5, 6].

Currently, there is no virus specific therapy available. Several antiviral agents have been studied in virus-infected cell lines in vitro. These agents include ribavirin, interferon alpha, and human immunoglobulin. Preliminary studies of ribavirin suggest that it inhibits the replication and cytopathogenicity of West Nile virus in human neural cells [5]. Interferon alpha reportedly protects spinal cord cells from becoming infected with the virus. One case reported from Israel described the successful use of immunoglobulin from pooled Israeli donors in a comatose West Nile encephalitis patient [5, 6].

There are no vaccines available at the moment. Several laboratories are conducting research for their possible development. Methods of prevention include eliminating mosquito breeding areas, limiting outdoor activities during peak mosquito biting periods, and wearing protective clothing, such as long sleeves and long pants. Multiple studies evaluating a variety of mosquito prevention strategies support the efficacy of DEET (N,N-diethyl-m-toluamide) containing repellants [2, 5]. These sprays should be applied to both exposed skin and clothing [5].

Given how West Nile has spread and that it is now located across the entire North American continent, physicians and other health care providers should consider West Nile infection in the differential diagnosis of causes of aseptic meningitis and encephalitis, obtain appropriate laboratory studies, and promptly report cases to state public health authorities [7]. State governments should establish abatement programs that will eliminate sources that allow for mosquito reproduction and harboring. The public needs to be given resources that educate them on what entails the disease caused by the West Nile virus, what the symptoms are, and, most importantly, what they can do to prevent themselves from becoming infected.
